# A Closer Look at Dowling-Degos Disease: A Case Report and Quantitative Assessment of Its Surface Texture Parameters

**DOI:** 10.7759/cureus.104128

**Published:** 2026-02-23

**Authors:** Catherine F Sollitto, Claire Wolinsky, Brian L Beatty

**Affiliations:** 1 College of Osteopathic Medicine, New York Institute of Technology, Old Westbury, USA; 2 Department of Dermatology, Mount Sinai Hospital, New York City, USA; 3 Department of Anatomy, College of Osteopathic Medicine, New York Institute of Technology, Old Westbury, USA

**Keywords:** diagnostic technology, dowling-degos disease, genodermatoses, skin texture, surface metrology, surface topography

## Abstract

Dowling-Degos disease (DDD) is a rare genodermatosis characterized by reticulated hyperpigmented macules and papules, yet its surface architecture has not been quantitatively described. In this case, we utilized 3D surface metrology to objectively characterize its surface texture. A shave biopsy from the inner thigh of a 54-year-old woman with clinically and histopathologically confirmed DDD was scanned using the S Neox optical profiler (Sensofar, Barcelona, Spain) at 20x and 50x magnification. Roughness parameters, including mean roughness (Sa), maximum surface height (Sz), maximum valley depth (Sv), maximum peak height (Sp), root mean square roughness (Sq), skewness (Ssk), and sharpness (Sku), were extracted and compared with previously published values for both unaffected and psoriatic skin. When compared to unaffected skin, DDD showed markedly increased Sa, Sz, and Sv, indicating a more irregular and deeply sculpted skin surface. In contrast to psoriatic lesions, DDD demonstrated lower Sa. Between magnifications, Sp was significantly greater in the 20x scan. These findings indicate that DDD has a distinct topographic profile that could support noninvasive diagnosis and monitoring. Surface metrology may complement clinical, dermoscopic, and histopathologic evaluation by providing a quantitative description of disease-specific skin texture.

## Introduction

Dowling-Degos disease (DDD) is a rare genodermatosis that most often affects flexural regions, including but not limited to the axillae, inframammary folds, and neck. Due to its autosomal-dominant pattern of inheritance, DDD frequently presents with a family history, although it may develop sporadically as well. DDD primarily manifests as benign hyperpigmented reticulated macules and papules during or shortly after puberty and may continue to progress throughout adulthood. Because of its characteristic presentation, standard diagnosis of DDD is usually clinical with dermoscopy and can be confirmed via histopathological analysis of a skin biopsy [1].

In a field where superficial features such as texture and morphology help to determine diagnoses, it is critical for dermatologists to have access to various techniques that can quantify such characteristics. Currently, clinical diagnosis via visual or tactile evaluation remains among the most cost-effective, convenient, and timely diagnostic methods. However, both tactile and visual sensation are subjective measures that allow room for diagnostic error [2]. Primarily, palpatory abilities vary amongst individuals; differing skill levels, experience, and sensory corpuscle distribution within the hand may influence clinical decision-making [3, 4]. Also, while visual evaluation can be assisted by a dermatoscope, palpation lacks similar modes of immediate assistance and uniformity. Moreover, palpation is not always feasible when there is potential for secondary infection, irritation, or pain elicitation [5, 6]. Thus, palpation as a diagnostic tool presents both advantages and setbacks, which introduces the need for more improved, consistent methods of textural evaluation.

Surface metrology offers an objective measurement of surface texture by capturing the complete geometrical structure of dermatoses, including textural direction, reflectance properties, and viewpoint. It also allows for quantitative measurement of disease progression, as it produces a point cloud that defines the surface in mathematical detail [7]. The images produced by surface metrology may also act as a visual tool that surpasses language or educational barriers when explaining complex dermatological conditions. Visualization of the actual skin texture would enable patients to better understand the physical nature of their condition, as opposed to histopathological images, which require further educational training to understand [8, 9].

Dowling-Degos disease has yet to be analyzed under surface metrology or quantified in terms of surface roughness or composition. In this case, we demonstrate the application of 3D surface metrology to better characterize its textural morphology and explore its potential role in future identification and diagnosis.

## Case presentation

**Figure 1:**
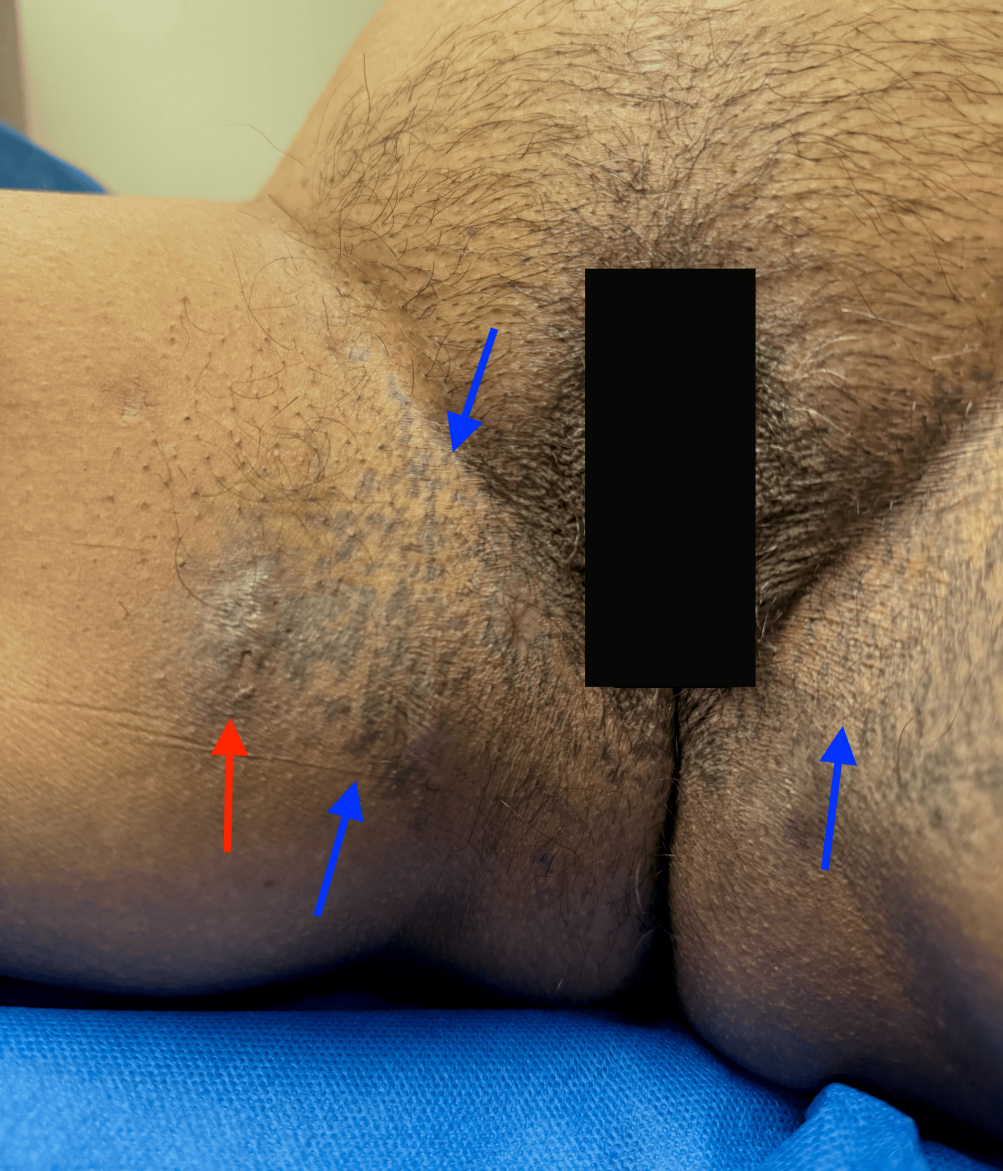
A shave biopsy was taken from the right inner thigh of the patient. Dowling-Degos disease is depicted on both the left and right inner thighs (blue arrows) and is accompanied by crusted hidradenitis suppurativa ulcers (red arrow).

A 54-year-old South Asian woman with a history of hidradenitis suppurativa (HS) presented to the hospital dermatology clinic with macular reticulate hyperpigmentation in the axillary, inguinal, and vulvar regions (Figure 1). The hyperpigmentation was accompanied by pigmented fistulae in her axillae and ulcers in the inguinal and vulvar regions. The patient denied a family history of either condition and stated that symptoms had only appeared within the past four years following radiation therapy for a right breast carcinoma. Otherwise, she had not undergone any topical or systemic therapies to treat her condition.

Two shave biopsies of the right inner thigh were collected, one of which was immediately sent for histopathological analysis, and the other was stored in a 10% formalin solution until it could be scanned for topographical analysis. The pathology report showed epidermal hyperplasia accompanied by dilated follicular infundibula, hyperpigmentation of basal keratinocytes, and increased melanophages. Based on the histological and clinical findings, a diagnosis of DDD was made.

**Figure 2:**
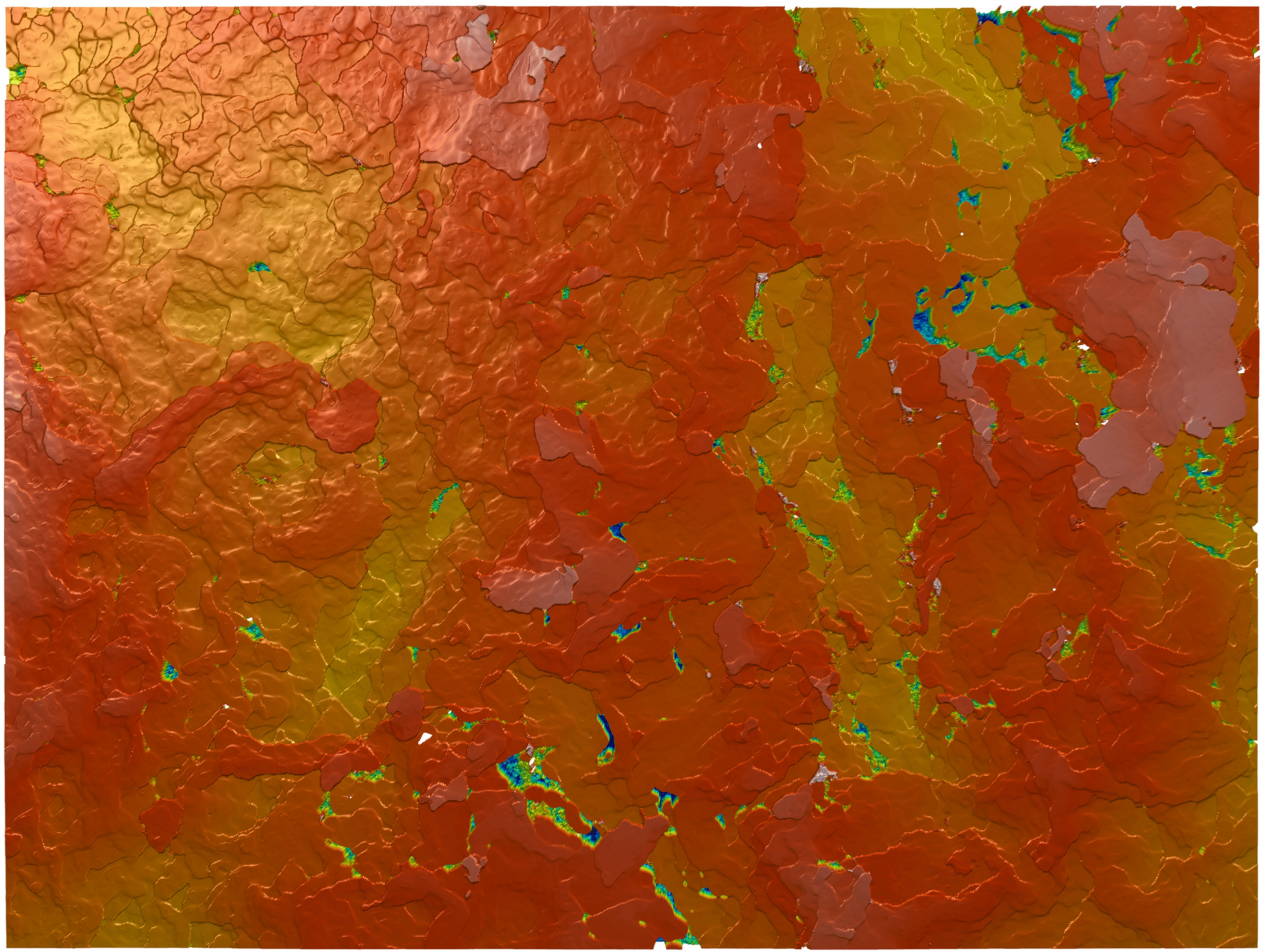
Surface topography of Dowling-Degos disease skin surface as depicted by a 20x scan The scan is positioned within the center of the skin sample and displays its various superficial peaks and valleys within the z-plane. Red denotes the highest peaks, followed by orange, yellow, and green. Blues denote the deepest valleys.

**Figure 3:**
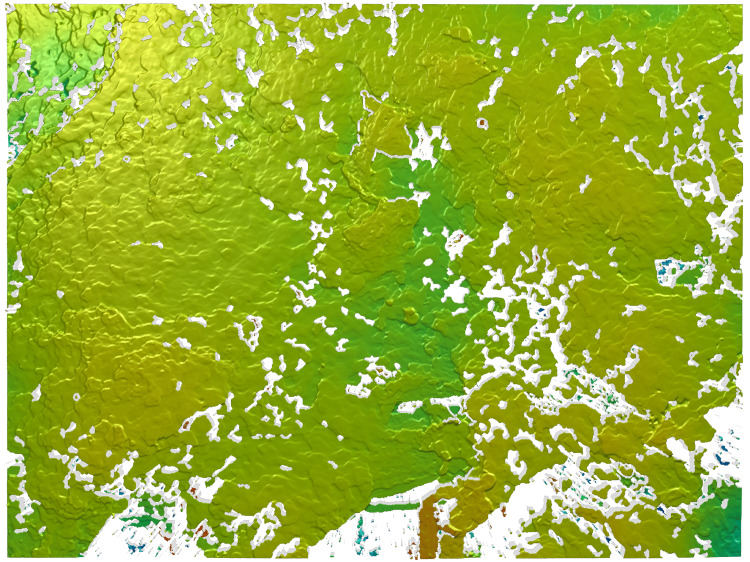
Surface topography of Dowling-Degos disease skin surface as depicted by a 50x scan The scan is positioned within the center of the skin sample and displays its various superficial peaks and valleys. The lighter green denotes more elevated areas, with darker green demonstrating deeper areas. Higher peaks are denoted with orange and red, seen at the bottom of the image.

The specimen was scanned with an S neox white light optical profiler (Sensofar, Barcelona, Spain) at the New York Institute of Technology College of Osteopathic Medicine Visualization Center (NYIT, Old Westbury, NY, USA). The sample was scanned in three separate regions at both 20x and 50x magnifications, yielding a scanned area of 873.33 x 656.61 𝜇m and 338 𝜇m x 283 𝜇m, respectively. The z-range was defined by the segment of the sample in focus. Both scans obtained data completeness of >99% (Figures 2, 3). A series of processing steps was completed, including leveling, area extraction, removing form (polynomial = 3), retouching, removing outliers, and repeat leveling. Data was extracted from each scan via ISO-25178-2 parameters, including mean roughness (Sa), maximum surface height (Sz), maximum valley depth (Sv), maximum peak height (Sp), root mean square roughness (Sq), skewness (Ssk), and sharpness (Sku) (Table 1).

**Table 1:** Comparison and explanation of surface roughness parameters yielded by 20x and 50x scans Paired t tests were used to compare the mean of each parameter between 20x and 50x scans. The parameters were as follows: mean height/roughness (Sa), maximum surface height (Sz), maximum peak height (Sp), root mean square roughness (Sq), skewness (Ssk), valley depth (Sv), and sharpness (Sku).

Parameter	Definition	Mean (20×) (µm)	Mean (50×) (µm)	Statistical Significance
Sa	Arithmetical mean height; represents average roughness of skin surface	33.48	8.08	No (p=0.060)
Sz	Maximum surface height; highlights distance between highest peak and deepest valley	340.07	237.30	No (p=0.370)
Sp	Maximum peak height; highest raised feature in measured surface	142.83	94.47	Yes (p=0.007)
Sq	Root mean square roughness; variability in height distribution	40.57	11.47	No (p=0.057)
Ssk	Skewness; describes asymmetry of height distribution	0.23	0.37	No (p=0.898)
Sv	Maximum valley depth; deepest depression on skin surface	198.93	142.83	No (p=0.578)
Sku	Kurtosis; indicates sharpness, scaling, spikes, or fissures	2.69	20.43	No (p=0.205)

Studies investigating the ISO-25178-2 roughness parameters of healthy, unaffected skin and psoriatic skin were compiled for comparison (Table 2). For studies where roughness was separated by age group, we extracted the data from the age group that encompassed that of our patient (54 years) for a more accurate comparison. When compared to the skin roughness parameters of unaffected individuals, both DDD scans displayed greater Sa, Sq, and Sz [10-12].

**Table 2:** Summary of studies reporting ISO 25178-2 surface roughness parameters in unaffected and psoriatic skin, used for comparison with Dowling-Degos disease skin. All findings values are presented in µm. Sa: mean height/roughness; Sz: maximum surface height (Sz); Sq: root mean square roughness

Study	Skin Condition	Findings
Askaruly et al. [10]	Unaffected forearm skin of individuals aged ~55 on average	Sa = 0.78 ± 0.28 Sq = 1.68 ± 0.67
Korn et al. [11]	Unaffected shoulder skin of individuals 40-59 years	Sa = 21.54 ± 4.79
Bloemen et al. [12]	Unaffected, “normal appearing” skin of various regions	Sa = 19.6 ± 5.4 Sz = 267 ± 90.3
Ahmad Fadzil et al. [13]	Psoriatic lesions of the head, trunk, upper and lower limbs across age groups	Sa (mild psoriasis, on average) = 24 Sa (severe psoriasis, on average) = 106 Sa range = 10-187

In contrast, psoriatic skin has been found to yield Sa measurements up to 187 𝜇m, versus 33.48 𝜇m (20x) and 8.08 𝜇m (50x) found in this study [13]. Mild psoriasis, however, generally has a lower Sa, making its texture more comparable to that of DDD (Table 2). Thus, our results display that DDD has unique textural characteristics that distinguish it from unaffected skin as well as other dermatologic conditions, thereby suggesting that it may be classified and diagnosed via these parameters.

When comparing the 20x and 50x magnifications, all paired differences were normally distributed (p > 0.05), thus paired t-tests were used throughout. Only Sp differed significantly between magnifications (p = 0.007), with higher peaks detected at 20x than 50x. Both Sa and Sq showed near-significant trends toward greater roughness at 20x, while others (Sz, Sv, Ssk, Sku) did not differ significantly (Table 1). These results reflect the fact that the 20x scan captures a more macroscopic, reticulated skin network (873.33 x 656.61 𝜇m vs. 338 𝜇m x 283 𝜇m), thus displaying much larger surface variations (Figures 2 and 3).

Written informed consent was obtained from the patient to publish the details of this case as well as the associated images.

## Discussion

In contrast to the typical earlier onset of DDD, our patient presented with disease onset in the sixth decade of life, with symptoms emerging after radiation therapy for breast cancer [14]. The clinical sequence raises the possibility that the patient’s breast cancer or its treatment contributed to disease onset; however, such an association has not been previously reported in the literature. The only comparable report of late-onset, sporadic DDD following radiation exposure involved ultraviolet A (UVA) therapy administered for psoriasis, which differs substantially from the high-energy, ionizing radiation administered for breast cancer treatment [15]. Other malignancies reported in association with DDD, including squamous cell carcinoma, keratoacanthoma, and melanoma, have been described only after the initial diagnosis of DDD and likely reflect a shared underlying follicular pathology [16-18].

The coexistence of HS and DDD seen in our patient supports prior evidence suggesting shared pathogenic mechanisms. Impaired NOTCH signaling has been implicated in both conditions, contributing to abnormal follicular keratinization and altered melanocyte and keratinocyte differentiation. Genetic studies have demonstrated that mutations in *PSENEN*, *POFUT1*, and *NCSTN *are often linked to shared cases of DDD with HS [19].

This study is the first of its kind to quantitatively characterize the surface texture of DDD. Historically, DDD has been defined by its clinical appearance and confirmatory histopathologic features. In our analysis, topographic scans demonstrated marked scaling, asymmetry, and significant variation in cutaneous surface height. These findings correlate with the underlying histopathologic architecture described in the pathology report, with intense height variations (Sa, Sz, Sp) likely due to the areas of epidermal hyperplasia and dilated infundibula (Table 1). These characteristics quantitatively distinguish DDD from both psoriasis and unaffected skin, with DDD demonstrating greater surface irregularity than normal skin and generally less pronounced roughness than psoriatic lesions (Table 2). Given that DDD includes three variants, with Galli-Galli disease frequently indistinguishable on clinical examination, additional studies are needed to evaluate whether surface metrology may assist in distinguishing these subtypes in a noninvasive manner (Table 3) [19, 20].

**Table 3:** Comparison of the three clinical variants of Dowling-Degos disease DDD: Dowling-Degos disease

Variant	Key Clinical Features	Typical Age of Onset	Family History	Histopathology
Classic DDD [14, 19]	Reticular brown macules in flexural areas (e.g., axillae, groin), comedo-like papules, pitted perioral scars	Puberty or early adulthood (3^rd ^to 4^th^ decades most common)	Autosomal dominant familial cases often reported	Epidermal hyperplasia, melanophage proliferation, elongated thin rete ridges with basilar pigmentation
Follicular DDD [21, 22]	Predominantly follicular lesions (papules) with less prominent reticulate pigmentation	2^nd^ to 4^th^ decades	Autosomal dominant	Similar to classic DDD but with prominent follicular hyperkeratosis and plugging
Galli-Galli Disease (Acantholytic variant) [19, 20]	Clinically indistinguishable from classic DDD but with additional acantholysis	Similar to classic DDD	Considered autosomal dominant	Same as classic DDD + nondyskeratotic acantholysis on histology

This technology offers several potential advantages for clinical research of DDD and other cutaneous diseases. The characteristic epidermal alterations of DDD are typically appreciated only through histopathologic evaluation, which requires an invasive biopsy procedure. In contrast, while histology evaluates cellular details, surface metrology noninvasively quantifies the palpable surface morphology, including measurable parameters such as roughness, depth, and contour irregularities. Incorporating surface metrology into the study of cutaneous disorders may therefore enable objective, reproducible assessment of skin texture and help define disease-specific surface patterns that could enhance diagnostic accuracy and longitudinal monitoring.

Although our sample underwent an ex vivo laboratory scanning process, similar tools such as GelSight® (GelSight, Waltham, USA) can be used to achieve in-office surface metrology measurements [23]. Like the S neox, GelSight objectively characterizes the physical skin topography and provides detailed, 3D surface texture measurements within seconds to minutes. The process of using GelSight in a clinical setting is quick, non-destructive, and may be completed by trained clinical staff: a handheld probe is placed briefly in contact with the area of interest, an image is captured, and quantitative surface measurements are generated. These measurements may be seen visually as a topographical heat map, which provides patients with a comprehensible, visual report of their skin, as in Figures 2 & 3, or as numerical values. The results are produced in real-time and are independent of lighting conditions or reflectivity, further differentiating it from both regular photography and histopathology [24]. In a clinical setting, this could allow for enhanced skin inspection, monitoring the severity of dermatoses, or assessing treatment efficacy. Various studies have also utilized this technology to investigate skin barrier function, irritation/irritants, degree of damage conveyed by aging or other environmental processes, and to measure the efficacy of products and procedures that claim to repair the skin [25].

A paramount limitation of this case report is its reliance on a single specimen. The rarity of DDD makes it difficult to obtain multiple samples; since its initial identification, there have been fewer than 50 documented cases in the literature [26]. Although this report provides textural characterization of a single presentation of classic DDD, the condition may manifest as any of its three recognized clinical variants. Thus, further research is required to further define and compare its other variants.

## Conclusions

Overall, the findings in this case report may enhance established diagnostic methods by providing a more objective, mathematical characterization of the disease rather than relying on the subjectivity of physician palpation or clinical assessment. The marked elevations in roughness, height variation, and valley depth relative to unaffected skin confirm that DDD possesses a distinct and measurable surface architecture. Differences observed across magnifications further highlight the multiscale complexity of the disease’s reticulated morphology. Although limited to a single specimen, these findings establish a foundational framework for future studies investigating larger cohorts and variant presentations. Continued application of quantitative surface analysis may ultimately support earlier and less invasive detection, monitoring, and improved differentiation of DDD from clinically similar conditions.
